# Systematic Analysis of the *Populus* ADF Gene Family and the Expression Patterns Under Osmotic Stress

**DOI:** 10.3390/life16050800

**Published:** 2026-05-11

**Authors:** Yanli Yang, Hailong An, Hui-Guang Li, Yuanlin Sun, Baozhen Feng, Peiqian Li

**Affiliations:** 1 Department of Life Sciences, Yuncheng University, Yuncheng 044000, China; 2 Shanxi Technology Innovation Center of High Value-Added Echelon Utilization of Premium Agro-Products, Yuncheng 044000, China; 3 Department of Resource Management, Tangshan Normal University, Tangshan 063000, China; 4 Institute of Horticultural Crops, Yunnan Academy of Agricultural Sciences, Kunming 650205, China

**Keywords:** ADF, *Populus*, gene duplication, gene expression, osmotic stress

## Abstract

Actin Depolymerizing Factor (ADF) proteins are key regulators of actin cytoskeleton dynamics, mediating numerous essential plant life processes, including cell elongation, division, and signal transduction in response to environmental stress. Although ADF functions are well characterized in herbaceous plants, systematic analysis of poplar ADFs and their roles in osmotic stress response remains largely unexplored. In this study, we identified 14 *PtADF* genes in the *Populus trichocarpa* genome, mapped across ten distinct chromosomes. Phylogenetic analysis categorized all the ADFs into seven groups, with PtADFs displaying conserved motifs. *PtADF* gene family expansion was primarily attributed to whole-genome duplication (WGD) events. Evolutionary constraint analysis, evidenced by a Ka/Ks ratio < 1, indicated significant selective pressure on these genes. Promoter regions of *PtADF* genes were enriched with *cis*-acting elements responsive to hormones and stresses. Transcriptome profiling showed that five *PtADF* genes were significantly induced under drought stress. We then identified the homologous genes of *PtADFs* in *P. euphratica*, a *Populus* species with superior environmental stress adaptability, and qRT-PCR analysis revealed that four homologous *PeADFs* were significantly induced by mannitol treatment. These results characterize the basic features of the PtADF gene family and provide a general reference for screening candidate *PeADF* genes for further research in poplar.

## 1. Introduction

Among the various abiotic stresses, water stress is a prevalent and significantly detrimental factor affecting plant health, particularly for sessile plants [1]. This situation has been further aggravated by global warming along with the increase in high-temperature weather events, which has led to higher water resource consumption [2]. The impacts caused by drought stress are multifaceted, encompassing oxidative damage, impaired growth and development, damage to membrane compounds and nucleic acids, reduced photosynthesis, metabolic disorder, and apoptosis [3]. Osmotic stress is a primary physiological consequence triggered by drought-induced water deficit. Under drought conditions, the decreased soil moisture upsets the water potential gradient across cell membranes, leading to cell dehydration, reduced cell turgor, and metabolic dysfunction [4,5]. Plants employ two strategies to cope with drought and osmotic stress. One strategy is minimizing water consumption by closing stomata, reducing growth rates, and inducing senescence or dormancy [6,7,8]; the other is enhancing water absorption by modifying root structures, utilizing hydrotropism, and adopting specialized C4 or CAM photosynthetic pathways [9,10,11]. Plants have evolved interrelated regulatory pathways enabling prompt response and adaptation to their environment [12]. They can quickly perceive stress signals and initiate the expression of stress-responsive genes, including various transcription factors and osmotic regulatory genes, which are involved in the synthesis of stress-related metabolites.

Actin, a principal constituent of the cytoskeleton, serves as a crucial factor in the cellular stress response. In plant cells, actin is present in two forms: globular actin (G-actin) and filamentous actin (F-actin). The actin depolymerizing factor (ADF/cofilin) is highly conserved across eukaryotic cells and plays a key role in regulating actin filament assembly and disassembly dynamics [13]. Research has demonstrated that *ADFs* influence plant growth and development and stress adaptation by responding to various physiological and stress signals [14]. In *Arabidopsis*, the genome encodes 11 ADF proteins, which can be categorized into four distinct subcategories (I-IV) [15]. The maize genome contains 13 *ADF* genes (*ZMADF1*–*ZMADF13*), among which three genes (*ZmADF2*, *ZmADF3*, and *ZmADF4*) show specific responses to drought stress [16]. In rice, eleven *OsADF* genes demonstrated varying expression patterns across different developmental stages and tissues, indicating different functions of these *OsADFs* in growth and reproduction. Overexpression of *OsADF3* enhanced primary root length, germination, and survival rates, thereby increasing tolerance to mannitol and drought stress in *Arabidopsis* [17]. Similarly, soybean GmADF13 takes a positive role in regulating drought stress by activating the expression of *GmbZIP1*, *GmDREB1A*, and *GmANK114* [18].

*Populus* has become a key species in addressing climate change due to its unique physiological adaptations, fast growth rate, and its role as an ecological indicator [19,20,21]. In the context of climate change, poplars are of vast potential in mitigating the greenhouse effect by making a significant contribution to carbon sequestration [19]. However, as an important tree species for ecological restoration and shelterbelts, the *ADF* genes and their roles in drought/osmotic resistance remain understudied in poplar, despite extensive progress having been made in other plant species. In this study, we systematically identified and characterized the ADF gene family in *P. trichocarpa* at a genome-wide level, including phylogeny, conserved motifs, domains, gene structures, chromosomal distribution, and collinearity. Based on transcriptome data, we further investigated the expression profiles of *PtADFs* under drought stress. In addition, as *P. euphratica* is an important poplar species with splendid adaptation ability to drought, high salinity, and osmotic stress, and it possesses great application value in forest genetics research and breeding [22,23]; we used *P. euphratica* as experimental material and performed qRT-PCR to analyze the expression patterns of candidate stress-responsive *PeADFs*. This study clarifies the characteristics of the *P. trichocarpa* ADF family, screens candidate *PeADFs* responsive to osmotic stress in *P. euphratica*, and lays a foundation for further functional research in poplar.

## 2. Materials and Methods

### 2.1. Plant Materials

Comparable 1-year-old *P. euphratica* seedlings were trimmed to 15 cm and cultivated in pots (30 cm × 20 cm) for 60 days. To simulate osmotic stress in *P. euphratica*, D-mannitol was used in accordance with well-established protocols from published poplar studies [24,25,26]. Slight experimental modifications were made with reference to three peer-reviewed studies on osmotic stress in woody plants, including two previous studies on *P. euphratica* [27,28] and one relevant study on apples [29]. Seedlings were carefully removed from the soil and hydroponically cultured in 10 L containers containing Hoagland nutrient solution supplemented with 300 mM D-mannitol. Roots were continuously aerated to maintain an adequate oxygen supply during the treatment. Leaf tissues were sampled at 0, 3, 9, 24, and 36 h after mannitol treatment and immediately frozen in liquid nitrogen and stored at −80 °C for RNA isolation.

### 2.2. Identification and Prediction of Physicochemical Properties of ADFs in Populus

Genomic data for *P*. *trichocarpa* were obtained from the Phytozome database (https://phytozome-next.jgi.doe.gov/, accessed on 12 March 2025). Homologs of *Arabidopsis thaliana ADF* genes were identified in the *P. trichocarpa* genome using their full-length amino acid sequences of AtADFs. Conserved protein domains of all the candidate PtADF sequences were further verified using the SMART database (http://smart.embl-heidelberg.de/, accessed on 18 March 2025). Physicochemical properties of the PtADFs, including amino acid sequence length, molecular weight, isoelectric point, and instability index, were analyzed with the ProtParam tool on the ExPASy server (https://web.expasy.org/protparam, accessed on 20 March 2025). Subcellular localization of the PtADF family was predicted using the Plant-mPLoc server (http://www.csbio.sjtu.edu.cn/bioinf/plant-multi/, accessed on 21 March 2025).

### 2.3. Construction of the Phylogenetic Tree

*A. thaliana* genomic data were sourced from the TAIR database (https://www.arabidopsis.org/), while the genomic sequences for *Salix purpurea*, *Oryza sativa*, and *Malus domestica* were obtained from the Phytozome database (https://phytozome-next.jgi.doe.gov/). The best-fit evolutionary model was selected using ModelTest-NG v0.1.7 [30], and a maximum likelihood (ML) phylogenetic tree was subsequently constructed using RAxML-NG v2.0.0 (https://github.com/amkozlov/raxml-ng, accessed on 16 April 2026) with a Bootstrap parameter set to 1000 [31]. The resulting tree was visualized and annotated using the ITOL online server (https://itol.embl.de/, accessed on 16 April 2026).

### 2.4. Chromosome Localization and Synteny Analysis

We used TBtools software v2.0.30 (https://github.com/CJ-Chen/TBtools-II/, accessed on 14 June 2025 ) to visualize the chromosomal locations of identified *PtADF* genes [32]. Intraspecific and interspecific collinearity analysis is crucial for the investigation of gene families [33]. The syntenic relationships among *PtADF* genes were analyzed using TBtools, and the Python implementation of MCScanX (JCVI v0.9.14) was employed to identify collinear blocks of *ADF* genes across multiple species, including *A. thaliana*, *Solanum lycopersicum*, *P. trichocarpa*, *O. sativa*, and *S. purpurea.* Gene duplication analysis was conducted utilizing DupGen finder with the default settings [34,35]. To assess evolutionary divergence among duplicated *PtADF* genes, TBtools was used to calculate synonymous (Ks) and nonsynonymous (Ka) substitution rates, as well as the Ka/Ks ratio for each gene pair [34].

### 2.5. Analysis of Conserved Motifs, Domains, and Gene Structures

Conserved motifs of PtADF proteins were identified using the MEME online server (http://meme-suite.org/), with the maximum number of conserved motifs set to 10. The NCBI Conserved Domain Database (CDD) was used to predict conserved structural domains (https://www.ncbi.nlm.nih.gov/Structure/cdd/wrpsb.cgi, accessed on 20 March 2025). The GFF3 annotation file of *P. trichocarpa* was used to analyze the exon–intron structure of *PtADF* genes. Conserved motifs, domains, and gene structures were visualized using TBtools.

### 2.6. Prediction of the Cis-Acting Elements of PtADF Promoters

The 2000 bp sequences upstream of the ATG start codon for all *PtADF* genes were extracted from the genome and defined as promoter regions. These promoter sequences were then submitted to the PlantCARE database (https://bioinformatics.psb.ugent.be/webtools/plantcare/htm, accessed on 5 July 2025) for *cis*-element prediction [36], and the results were visualized using TBtools.

### 2.7. Expression Pattern Analysis

To investigate the expression patterns of *PtADFs* under various water stress conditions, we downloaded publicly available RNA-seq data from the NCBI database (project accession: PRJEB19784). Transcriptome data corresponding to short-term and prolonged drought treatments were extracted to characterize the stress-responsive expression of identified PtADF family members. Subsequent data normalization and heatmap visualization were performed using R software (v. 4.3.2; https://www.r-project.org/) to illustrate the expression patterns of *PtADF* genes under drought conditions.

### 2.8. RNA Extraction and qRT-PCR Analysis

For each treatment, five healthy *P. euphratica* seedlings with uniform growth status were selected. After treatment, leaf tissues were harvested. Total RNA from *P. euphratica* leaves was extracted using a Plant Total RNA Extraction Kit (Aidlabs Bio Inc., Beijing, China). RNA quality and concentration were evaluated using a NanoDrop 2000 Spectrophotometer (Thermo Fisher Scientific, Waltham, MA, USA). Subsequently, 2 μg of total RNA from each sample was used for cDNA synthesis with the Fast Quant RT Kit (Tiangen Biotech Co., Ltd., Beijing, China) following the manufacturer’s guidelines. Quantitative real-time PCR assays were performed as previously described [37]. We conducted the assay using SuperStar Universal SYBR Master Mix (Beijing CoWin Biotech Co., Ltd. (Beijing, China)), on a CFX96 real-time PCR detection system (Bio-Rad, Hercules, CA, USA). The qRT-PCR thermal cycling conditions were as follows: an initial incubation at 95 °C for 3 min, followed by 40 cycles of denaturation at 95 °C for 15 s and annealing at 60 °C for 15 s. Relative gene expression levels were calculated using the 2^−ΔΔCt^ method, with poplar 18S rRNA serving as the internal reference gene for normalization, as described previously [27,37,38,39]. For qRT-PCR, leaf tissues from five individual seedlings were pooled into a single composite sample for each treatment group. Three technical replicates were set for each qRT-PCR reaction. The primers used in this study are listed in Supplementary Table S1.

### 2.9. Statistical Analysis

All experimental data are presented as mean ± SD. Different lowercase letters indicate significant differences based on one-way ANOVA and Tukey’s test (*p* < 0.05) [40,41]. Normality and homogeneity of variance were verified before ANOVA. One-way ANOVA was used to detect overall expression differences. Tukey’s post hoc test was applied to compare each D-mannitol treatment time point with the 0 h control.

## 3. Results

### 3.1. Identification of PtADFs in Poplar

**Figure 1:**
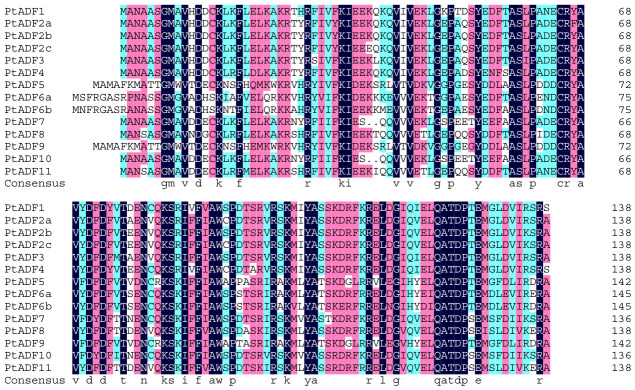
Amino acid sequence alignment of the PtADFs protein of poplar trees (*Populus trichocarpa*).

Based on the integration of identification results from NCBI online BLAST and verification results from SMART analysis, we ultimately determined that the genome of *P*. *trichocarpa* encodes 14 ADF family members (designated as PtADFs). These 14 *PtADF* members were named in correspondence with their homologous *ADF* genes in *A*. *thaliana* (*AtADFs*) (Table 1). Among these PtADF proteins, the length of amino acid sequences ranged from 137 to 146 residues, with molecular weights varying from 15.792 kDa to 16.844 kDa. The theoretical isoelectric points (pI) of these proteins ranged from 5.11 to 8.41, and their instability indices were between 28.62 and 59.68 (Table 1). Subcellular localization prediction indicated that all 14 PtADF proteins are localized in the cytoplasm (Table 1). Additionally, multiple sequence alignment revealed that the amino acid sequences of all the PtADF proteins share a high similarity of 77.94% (Figure 1).

### 3.2. Phylogenetic Analysis of the ADF Family Members

**Figure 2:**
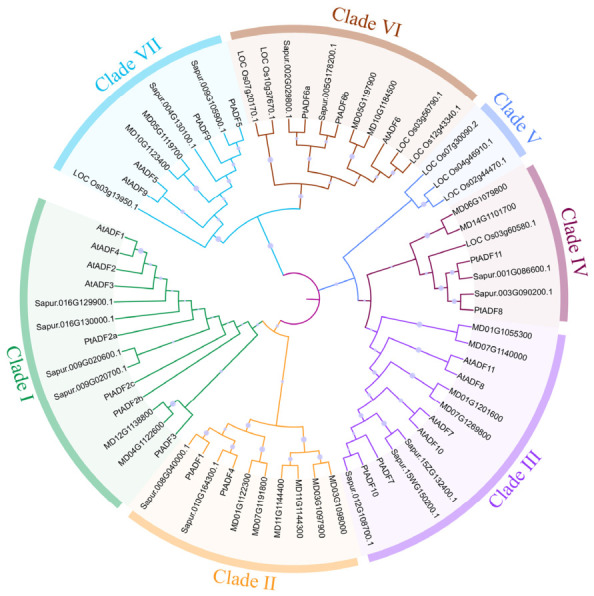
A phylogenetic analysis of the plant ADF gene family across *P. trichocarpa*, *A*. *thaliana*, *S*. *purpurea*, *O*. *sativa*, and *M*. *domestica*.

To investigate the evolutionary relationships among the *PtADF* gene family of *P. trichocarpa*, a phylogenetic tree was constructed using the maximum likelihood (ML) approach together with the ADFs of *A. thaliana*, *S. purpurea*, *O.sativa*, and *M. domestica*. As illustrated in Figure 2, the ADF proteins could be divided into seven distinct clades. Among them, PtADF members were distributed across six clades. Clade I included AtADF1-4, and PtADF2a, PtADF2b, PtADF2c, and PtADF3. Clade II comprised PtADF1 and PtADF4. Clade III and Clade IV had a relatively close relationship, harboring AtADF7-8, AtADF10-11, as well as PtADF7, PtADF10, PtADF8, and PtADF11. Clade VI consisted of 11 members, dominated by ADF6 homologs containing PtADF6a and PtADF6b. In Clade VII, PtADF5 exhibited homology with AtADF5, implying potentially conserved functions. Additionally, AtADF9 and PtADF9 were also assigned to Clade VII.

### 3.3. Chromosomal Spread and Duplication Event Analysis of the PtADF Genes

**Figure 3:**
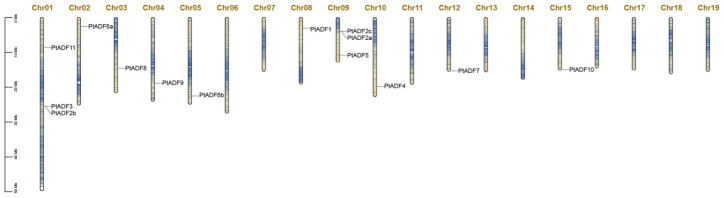
Chromosomal locations of *PtADFs* in poplar. The lengths and gene densities of the 19 chromosomes were represented by bands. Blue lines indicated relatively low gene densities.

**Figure 4:**
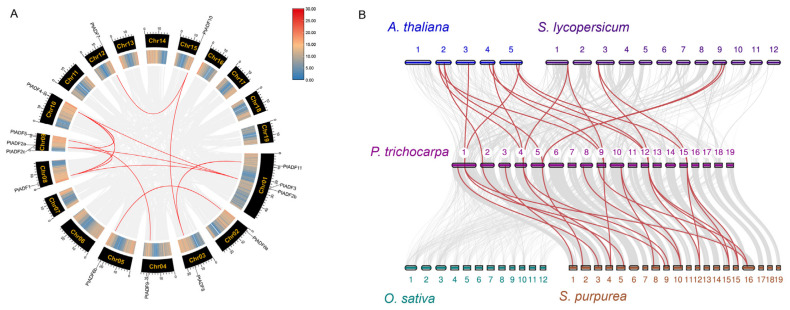
Syntenic analysis of the ADF gene family. (**A**) Intra-chromosomal synteny of *ADF* genes in *P. trichocarpa*. Red lines represent collinear gene pairs of PtADF members, and the legend indicates gene density across each chromosome. (**B**) Cross-species synteny comparison of *ADF* genes between *P. trichocarpa* and four other plant species, including *A. thaliana*, *S. lycopersicum*, *O. sativa*, and *S. purpurea*. Gray lines denote conserved syntenic blocks between pairs of genomes, whereas red lines highlight the syntenic ADF homologous gene pairs.

All the PtADFs were successfully mapped to the linkage groups of the *P. trichocarpa* genome, exhibiting an uneven distribution across 10 chromosomes (Figure 3). Chromosomes 1 and 9 harbor the highest number of *PtADF* genes, with three members distributed on each. In contrast, only one *PtADF* gene is located on chromosomes 2, 3, 4, 5, 8, 10, 12, and 15. Gene duplication serves as a significant source of genetic material that drives species adaptation and diversification in plants [42,43]. We identified 11 syntenic gene pairs within the PtADF family (Figure 4A). To further investigate the evolutionary patterns of PtADFs, we calculated the Ka, Ks, and Ka/Ks ratios for these duplicated gene pairs. All pairs showed a Ka/Ks ratio less than 1 (Table 2), suggesting that the PtADF family has undergone strong purifying selection throughout the course of evolution. For comprehensively elucidating the phylogenetic mechanisms of PtADFs, we analyzed the synteny between *P. trichocarpa* and four additional species, including *A. thaliana*, *S. lycopersicum*, *O. sativa*, and *S. purpurea*. The results indicated that PtADFs exhibited close origin relationships with *S. purpurea*, *S. lycopersicum*, and *A. thaliana*. By contrast, PtADFs demonstrated a relatively distant genetic relationship with *O. sativa* (Figure 4B).

### 3.4. Structural Characterization of the PtADF Genes

**Figure 5:**
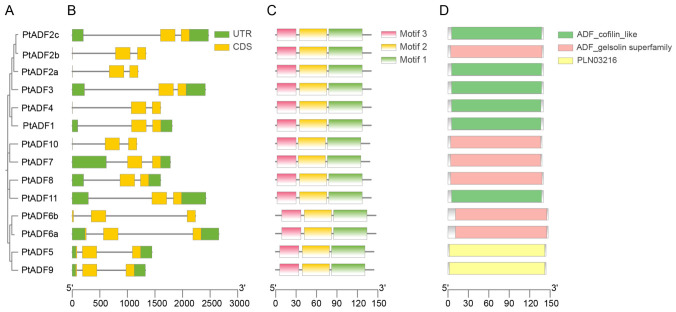
Phylogenetic relationships, gene structures, motif distribution, and domains of the *PtADF* genes. (**A**) Phylogenetic tree constructed from PtADFs. (**B**) Exon–intron structures of the *PtADF* genes. Green boxes and black lines represent the UTRs and introns, respectively. (**C**) Motif distribution of the PtADF members. (**D**) Conserved domain analysis of the PtADF proteins. Different domains are indicated in various colors.

To elucidate the evolutionary relationships among various members of the *PtADF* gene family, a phylogenetic tree was constructed based on the 14 PtADF protein sequences (Figure 5A). We then analyzed their gene structures, conserved protein motifs, and conserved domains. Structural characterization showed that all *PtADF* members harbored three exons and two introns (Figure 5A). Meanwhile, three conserved motifs were identified in PtADF proteins via the MEME server (Figure 5C). These motifs were widely distributed across all the PtADFs, indicating that they are highly conserved during evolution. In addition, we identified three conserved domains (ADF gelsolin superfamily, PLN03216, and ADF cofilin_like) in the PtADF proteins using the CDD (Figure 5D). Among the analyzed PtADF proteins, six contained an ADF_cofilin_like domain, six contained the ADF_gelsolin superfamily domain, and the remaining two possessed the PLN03216 conserved domain (Figure 5D).

### 3.5. Promoter Cis-Acting Elements Analysis of the PtADF Genes

**Figure 6:**
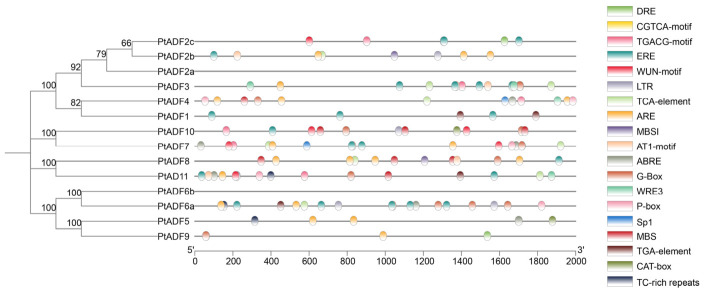
Annotation and distribution of the *cis*-elements in the *PtADFs* promoters. Different colors indicated different *cis*-acting elements, including stress and growth-related *cis*-elements.

The *cis*-acting elements in the promoters of *PtADF* genes were analyzed using the PlantCARE database (Figure 6). A variety of *cis*-acting elements related to growth and development were identified, including meristem expression elements (CAT-box), light-responsive elements (Sp1, AT1-motif, G-box), gibberellin-responsive elements (P-box), and auxin-responsive elements (TGA-element), among others (Figure 6). For instance, auxin-responsive elements were detected in the *PtADF1* promoter, and P-box elements were found in the promoter region of *PtADF4*, implying that the transcription of these genes may be regulated by the corresponding phytohormones. Similarly, elements related to flavonoid biosynthesis were identified in the promoters of *PtADF2b* and *PtADF8*.

Moreover, abundant stress-related *cis*-elements were identified in the promoter regions of *PtADFs*, including those associated with biotic stresses (salicylic acid and MeJA responsiveness) and abiotic stresses (low temperature, anaerobic induction, dehydration, and salt stress). For example, the MBS element, an MYB-binding site associated with drought-inducible gene expression, was identified in the promoters of *PtADF8* and *PtADF10*. PtADF5 contained defense- and stress-responsive elements including ABRE in its promoter (Figure 6).

### 3.6. Expression Profiles of the Poplar ADFs Under Various Water Stress Conditions

**Figure 7:**
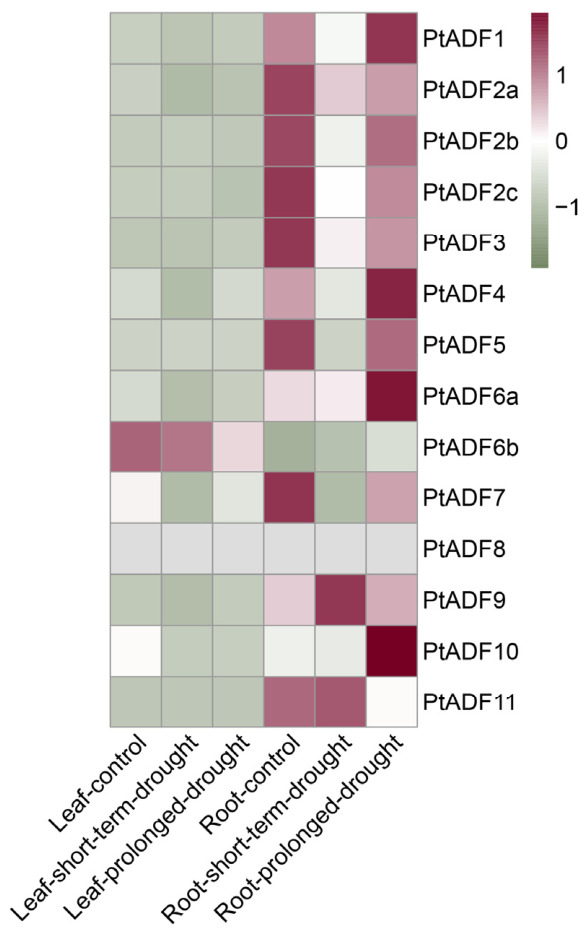
The heatmap illustrates the expression profile of poplar *PtADF* genes under water stress. Different colors indicate the relative transcript abundance of each gene.

Plants’ life processes are closely linked to the functions of the cytoskeleton. Microfilaments, which are primarily composed of actin, are a crucial component of the cytoskeleton. An increasing number of studies have demonstrated that ADFs/cofilin play roles in stress responses [44]. Expression analysis of 14 *PtADF* genes under drought stress revealed five upregulated members (Figure 7). Specifically, *PtADF4*, *PtADF6a*, *PtADF6b*, *PtADF9*, and *PtADF10* were induced in roots under prolonged drought stress. The expression of *PtADF6b* and *PtADF9* in roots was also activated by short-term drought stress. Notably, *PtADF9* was upregulated in both leaves and roots in response to drought, with higher expression levels detected in leaves under prolonged drought stress (Figure 7). In roots, the expression of *PtADF2a*/*2b*/*2c*, *PtADF3*, and *PtADF7* was downregulated under both short-term and prolonged drought stress. Meanwhile, *PtADF11* showed an obvious reduction in expression under prolonged drought stress (Figure 7). In leaves, the expression of *PtADF2a*/*2b*/*2c* and *PtADF7* was repressed by prolonged drought stress, whereas the transcript level of *PtADF3* decreased under short-term drought stress (Figure 7).

**Figure 8:**
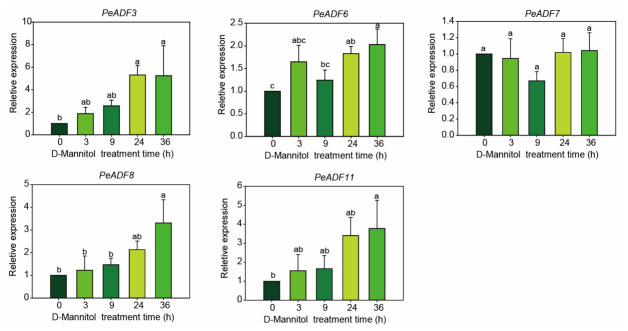
Expression levels of *P. euphratica ADF* genes under osmotic stress (D-mannitol treatment) detected by qRT-PCR. Different lowercase letters indicate significant differences at *p* < 0.05 (ANOVA followed by Tukey’s multiple comparison test).

Given its strong adaptability to extreme environments, *P*. *euphratica*, a typical desert riparian tree with exceptional drought resistance, serves as an excellent woody model for identifying stress-tolerance genes. Combined with the transcriptome expression profiles of *P. trichocarpa* (Figure 7), five upregulated *PtADF* genes were initially selected to screen their orthologous genes in *P. euphratica*. Based on the phylogenetic relationships of ADFs between the two poplar species (Figure S1), four definite orthologous pairs were identified: *PtADF4*, *PtADF6a*, *PtADF6b*, and *PtADF9* corresponded to *PeADF7*, *PeADF8*, *PeADF6*, and *PeADF3* in *P. euphratica*, respectively. *PtADF10* lacked an orthologous gene in *P. euphratica* and thus was excluded from subsequent analysis. Since *PtADF9* was upregulated in both leaves and roots under drought (Figure 7), we further analyzed *PeADF11* via qRT-PCR. *PeADF11* is an ortholog of *PtADF5* and clusters in the same phylogenetic clade as *PtADF9.* As shown in Figure 8, *PeADF8* was significantly upregulated at 36 h under mannitol treatment. *PeADF6* and *PeADF3* were significantly upregulated at 24 h and 36 h after mannitol treatment. *PeADF7* exhibited a slight downward trend at 9 h compared with the control. *PeADF11*, which clustered in the same clade as *PeADF3*, also showed a remarkable increase, with expression levels approximately 3.8-fold higher at 36 h. Overall, *PeADF3* showed the strongest induction trend, followed by *PeADF11*, *PeADF8*, and *PeADF6*. These data revealed distinct osmotic stress-responsive expression patterns of the five *PeADF* genes in *P. euphratica* leaves. Some genes showed pronounced induction, while one individual gene exhibited a weak response.

## 4. Discussion

ADFs are highly conserved actin-binding proteins that modulate F-actin dynamics and regulate plant growth and development [14]. Angiosperms harbor numerous *ADF* genes, and genome-wide identification of the ADF family has been widely reported in higher plants [44]. Although 14 poplar ADFs have been reported previously [45], a systematic genome-wide analysis remains lacking. Here, 14 *PtADF* genes were identified, with a similar number reported in other plants: 11 in tomato [46], 11 in *Mimulus guttatus* [45], 13 in maize [16], and 9 in *Medicago sativa* [47]. Conserved ADF_gelsolin or ADF_cofilin_like domains were also detected in PtADF proteins (Figure 5D). Tandem and segmental duplications are major drivers of gene family expansion during evolution. Segmental duplication occurs more frequently owing to repetitive chromosomal regions [48,49]. Whole-genome duplication (WGD) is widespread in angiosperm evolution and improves environmental adaptability [50]. Our results show that WGD events substantially drove *PtADF* family evolution (Table 2; Figure 4A), in agreement with earlier findings. The genus *Populus* underwent a recent WGD event, and duplicated genes derived from this event occupy a large proportion of the poplar genome [51]. However, segmental duplications have been reported in the ADF families of alfalfa, wheat, tomato and maize, whereas no tandem duplication was detected [16,46,47,52]. Furthermore, all duplicated gene pairs in *P. trichocarpa* had Ka/Ks ratios < 1 (Table 2), revealing strong purifying selection for the *PtADF* family. Such strong purifying selection maintains the functional stability of duplicated genes throughout evolution.

ADF proteins are generally short. PtADFs consist of 137–146 amino acids, with molecular weights ranging from 15.792 kDa to 16.844 kDa (Table 1). These characteristics are more consistent with findings in alfalfa and tomato than with those in wheat [46,47,52]. Phylogenetic analysis and conserved motif analysis revealed that closely related *PtADF* gene pairs (*PtADF8*/*PtADF11* and *PtADF5*/*PtADF9*) shared identical conserved motifs (Figure 5). The phylogenetic tree and synteny analysis in *P. trichocarpa* revealed that *PtADF* genes share a closer genetic relationship with *S. purpurea* than with rice (Figure 4). This observation may be attributed to the fact that both poplar (*Populus*) and willow (*Salix*) belong to the *Salicaceae* family [53].

Research has demonstrated the vital roles of ADFs in plant stress responses [14]. Numerous stress and hormone-responsive *cis-*elements were detected in *PtADF* promoters (Figure 6). It is well documented that hormones regulate plant stress responses [5,54]. Hormone-responsive *cis*-elements in *PtADF* promoters suggest that *PtADF* expression may be regulated by diverse endogenous and exogenous stimuli. *ADF4* significantly enhanced drought resistance in *Arabidopsis* via CARK3-mediated phosphorylation regulation [55]. Similarly, SaADF2 maintains actin filament stability to improve drought tolerance [56]. AtADF7 acted as a positive regulator of osmotic tolerance by inhibiting VLN1, thereby modulating F-actin dynamics in root hairs under osmotic stress [57]. In this study, drought stress induced the expression of *PtADF4*, *PtADF6a*, *PtADF6b*, *PtADF9*, and *PtADF10* (Figure 7). These stress-responsive *PtADF* genes were used to screen homologous genes in *P. euphratica*, and one phylogenetically related gene was additionally selected for expression analysis (Figure S1). Four *PeADF* genes were induced by mannitol, while *PeADF7* displayed a slight downward trend (Figure 8). Notably, the expression pattern of *PeADF7* (putative ortholog of *PtADF4*) was not fully consistent with its *P. trichocarpa* ortholog in the RNA-seq data (Figure 7 and Figure 8 and Figure S1). This discrepancy may arise from multiple factors, although orthologous relationships between the two species were well-supported by the phylogenetic tree (Figure S1). In *P. trichocarpa*, *PtADF4* expression was reduced in leaves under short- and prolonged drought, and elevated in roots under prolonged drought. *PeADF7* also tended to decrease in leaves under mannitol treatment (Figure 7 and Figure 8), suggesting strong tissue-specific regulation of poplar. Tissue-specific expression patterns of ADF homologs also widely exist in other plant species. Subclass III *AtADF5* and *AtADF9* exhibit weak expression in vegetative tissues, while subclass IV *AtADF6* is moderately expressed in various tissues. In particular, *AtADF9* is highly accumulated in root subapical regions, trichomes and callus [15], and *AtADF5* is specifically expressed in root tip meristems [58]. In addition, *ZmADF5* presents moderate expression in maize leaves [16]. In wheat, the expression of *TaADF* varied among different tissues at the same growth stage [52]. These results suggest that *ADF* gene expression varies among species, which may reflect functional specialization in different tissues. However, cross-species comparisons are constrained by inherent differences in drought adaptation, distinct stress treatments (soil drought vs. D-mannitol simulation), and potential regulatory divergence between orthologs. Accordingly, cross-species expression inferences should be made with caution.

Beyond varied tissue expression, *ADF* genes across subclades exert distinct regulatory roles in development and stress adaptation. AtADF6 acts as a negative regulator of plant resistance to powdery mildew by inhibiting RPW8.2 [59]. Similarly, GhADF6 negatively regulates resistance to *Verticillium dahliae* in cotton [60]. TaADF14/20, which belongs to the same clade as AtADF6, showed a decreased expression following freezing treatment [52]. In maize, ZmADF6/9/11, which has a closer genetic relationship with ADF6, showed increased expression levels responding to drought and heat stress [16]. Although ADF5 and ADF9 belong to the same subgroup, only ADF5 is critical for mature pollen function [61], and ADF9 is involved in pavement cell morphogenesis and is negatively regulated by MYB52 [62]. Another report showed that *AtADF5* mediates actin cytoskeleton remodeling during ABA-induced stomatal closure, contributing to drought tolerance [63]. In our study, the ADF5/9 orthologs in *P. euphratica*, *PeADF11*/*3*, were upregulated under mannitol treatment (Figure 8), consistent with drought-induced expression of maize *ZmADF5/8* [16]. Building on these findings, future work will explore their expression patterns under natural drought and investigate whether ABA may participate in their regulatory mechanisms. This study focuses on *PeADF* expression responses to osmotic stress. Meanwhile, given that plant water status assessment is critical for drought research, systematic water physiological measurements will be performed in further studies.

## 5. Conclusions

In this study, we conducted a genome-wide identification and comprehensive analysis of the *ADF* gene family in *P*. *trichocarpa*. A total of 14 *PtADF* genes were identified and unevenly distributed across ten chromosomes, with systematic characterization of their physicochemical properties, gene structures, phylogeny, and *cis*-regulatory elements. Promoter analysis revealed numerous stress- and hormone-related *cis*-elements, suggesting that *PtADF* genes may be potentially regulated at the transcriptional level under stress conditions. Expression analysis revealed that four *PeADF* members were induced under osmotic stress. Combined with bioinformatic findings, these expression data provide preliminary candidate genes for further stress-related research in poplar.

## Figures and Tables

**Table 1:** Detailed information on the *PtADF* genes.

Gene Name	Gene ID	Protein Length/aa	Isoelectric Point (PI)	Protein Molecular Mass/kDa	Instability Index	Predicted Subcellular Locations
*PtADF1*	Potri.008G052100.2	139	7.67	16.071	51.84	Cytoplasm
*PtADF2a*	Potri.009G028200.1	139	6.60	15.99	46.73	Cytoplasm
*PtADF2b*	Potri.001G236700.2	139	5.92	16.020	49.62	Cytoplasm
*PtADF2c*	Potri.009G028100.4	139	5.30	16.037	49.87	Cytoplasm
*PtADF3*	Potri.001G236400.2	139	6.60	15.945	51.87	Cytoplasm
*PtADF4*	Potri.010G208500.2	139	5.91	16.047	53.43	Cytoplasm
*PtADF5*	Potri.009G133100.1	143	8.41	16.514	36.39	Cytoplasm
*PtADF6a*	Potri.002G038800.1	146	7.75	16.829	39.57	Cytoplasm
*PtADF6b*	Potri.005G223800.2	146	6.84	16.844	40.76	Cytoplasm
*PtADF7*	Potri.012G141600.1	137	5.13	15.792	52.81	Cytoplasm
*PtADF8*	Potri.003G125500.1	139	5.26	15.960	54.20	Cytoplasm
*PtADF9*	Potri.004G173800.1	143	7.70	16.474	28.62	Cytoplasm
*PtADF10*	Potri.015G144500.1	137	5.13	15.804	55.62	Cytoplasm
*PtADF11*	Potri.001G106200.1	139	5.11	16.096	59.68	Cytoplasm

**Table 2:** Ka/Ks analysis and Gene duplication type of the orthologous *PtADF* genes. WGD: whole-genome duplication.

Gene Pairs	Ka	Ks	Ka/Ks	Selective Type	Gene Duplication Type
PtADF11-PtADF8	0.030996	0.522718	0.059298	Purifying	WGD
PtADF3-PtADF1	0.063716	0.980065	0.065012	Purifying	WGD
PtADF3-PtADF2c	0.031165	0.405347	0.076885	Purifying	WGD
PtADF3-PtADF4	0.063766	0.762537	0.083624	Purifying	WGD
PtADF6a-PtADF6b	0.05493	0.342636	0.160317	Purifying	WGD
PtADF8-PtADF10	0.157435	1.934172	0.081396	Purifying	WGD
PtADF9-PtADF5	0.018096	0.382673	0.047288	Purifying	WGD
PtADF1-PtADF2a	0.066852	1.459433	0.045807	Purifying	WGD
PtADF1-PtADF4	0.04073	0.220218	0.184955	Purifying	WGD
PtADF2c-PtADF4	0.056974	1.446886	0.039377	Purifying	WGD
PtADF7-PtADF10	0.014056	0.279598	0.050271	Purifying	WGD

## Data Availability

The publicly available RNA-seq dataset analyzed in this study was retrieved from the NCBI database with the project accession PRJEB19784. Original qRT-PCR data are listed in Table S2. All other data supporting the findings of this study are included within the article.

## Supplementary Materials

The following supporting information can be downloaded at https://www.mdpi.com/article/10.3390/life16050800/s1, Table S1: Primers used in this study. Table S2: qRT-PCR supplementary data. Figure S1: Phylogenetic analysis of *ADF* family genes in *Populus trichocarpa* and *Populus euphratica*.

## References

[1] Zhu J.K. (2016). Abiotic Stress Signaling and Responses in Plants. Cell.

[2] Gupta A., Rico-Medina A., Caño-Delgado A.I. (2020). The Physiology of Plant Responses to Drought. Science.

[3] Kim J.-S., Kidokoro S., Yamaguchi-Shinozaki K., Shinozaki K. (2024). Regulatory Networks in Plant Responses to Drought and Cold Stress. Plant Physiol..

[4] Yu B., Chao D.-Y., Zhao Y. (2024). How Plants Sense and Respond to Osmotic Stress. J. Integr. Plant Biol..

[5] Waadt R., Seller C.A., Hsu P.-K., Takahashi Y., Munemasa S., Schroeder J.I. (2022). Plant Hormone Regulation of Abiotic Stress Responses. Nat. Rev. Mol. Cell Biol..

[6] Zhang H., Zhu J., Gong Z., Zhu J.K. (2022). Abiotic Stress Responses in Plants. Nat. Rev. Genet..

[7] Guo C., Zhang K., Sun H., Zhu L., Zhang Y., Wang G., Li A., Bai Z., Liu L., Li C. (2025). Root Cortical Senescence Enhances Drought Tolerance in Cotton. Plant Cell Environ..

[8] Sun H., Yan L., Li Z., Cheng W., Lu R., Xia X., Ping J., Bian C., Wei N., You C. (2024). Drought Shortens Subtropical Understory Growing Season by Advancing Leaf Senescence. Glob. Change Biol..

[9] Zhang Y., Wu X., Wang X., Dai M., Peng Y. (2025). Crop Root System Architecture in Drought Response. J. Genet. Genom..

[10] Bailey-Serres J., Parker J.E., Ainsworth E.A., Oldroyd G.E.D., Schroeder J.I. (2019). Genetic Strategies for Improving Crop Yields. Nature.

[11] Zahedi S.M., Karimi M., Venditti A. (2021). Plants Adapted to Arid Areas: Specialized Metabolites. Nat. Prod. Res..

[12] Suzuki N., Rivero R.M., Shulaev V., Blumwald E., Mittler R. (2014). Abiotic and Biotic Stress Combinations. New Phytol..

[13] Maciver S.K., Hussey P.J. (2002). The ADF/Cofilin Family: Actin-Remodeling Proteins. Genome Biol..

[14] Inada N. (2017). Plant Actin Depolymerizing Factor: Actin Microfilament Disassembly and More. J. Plant Res..

[15] Ruzicka D.R., Kandasamy M.K., McKinney E.C., Burgos-Rivera B., Meagher R.B. (2007). The Ancient Subclasses of *Arabidopsis ACTIN DEPOLYMERIZING FACTOR* Genes Exhibit Novel and Differential Expression. Plant J..

[16] Huang J., Sun W., Ren J., Yang R., Fan J., Li Y., Wang X., Joseph S., Deng W., Zhai L. (2020). Genome-Wide Identification and Characterization of Actin-Depolymerizing Factor (ADF) Family Genes and Expression Analysis of Responses to Various Stresses in *Zea Mays* L. Int. J. Mol. Sci..

[17] Huang Y.C., Huang W.L., Hong C.Y., Lur H.S., Chang M.C. (2012). Comprehensive Analysis of Differentially Expressed Rice Actin Depolymerizing Factor Gene Family and Heterologous Overexpression of *OsADF3* Confers *Arabidopsis thaliana* Drought Tolerance. Rice.

[18] Wang D., Du M., Lyu P., Li J., Meng H., Liu X., Shi M., Gong Y., Sha Q., Men Q. (2024). Functional Characterization of the Soybean Glycine Max Actin Depolymerization Factor GmADF13 for Plant Resistance to Drought Stress. Plants.

[19] Fiege C., Germer S., Schwarz A., Bischoff W.-A., Pecenka R. (2025). Coarser Root Residues Are a Major Subsoil Carbon Sink after Re-Conversion of Poplar Short Rotation Coppice Plantation to Cropland. Biomass Bioenergy.

[20] Rosso L., Cantamessa S., Bergante S., Biselli C., Fricano A., Chiarabaglio P.M., Gennaro M., Nervo G., Secchi F., Carra A. (2023). Responses to Drought Stress in Poplar: What Do We Know and What Can We Learn?. Life.

[21] Guan P., Zheng Y., Lei G., Liu Y., Zhu L., Guo Y., Wang Y., Xi B. (2022). Near-Earth Remote Sensing Images Used to Determine the Phenological Characteristics of the Canopy of *Populus tomentosa* B301 under Three Methods of Irrigation. Remote Sens..

[22] Gai Z., Zhai J., Chen X., Jiao P., Zhang S., Sun J., Qin R., Liu H., Wu Z., Li Z. (2021). Phylogeography Reveals Geographic and Environmental Factors Driving Genetic Differentiation of *Populus* sect. Turanga in Northwest China. Front. Plant Sci..

[23] Brosché M., Vinocur B., Alatalo E.R., Lamminmäki A., Teichmann T., Ottow E.A., Djilianov D., Afif D., Bogeat-Triboulot M.-B., Altman A. (2005). Gene Expression and Metabolite Profiling of *Populus euphratica* Growing in the Negev Desert. Genome Biol..

[24] Ibáñez C., Vergara A., Castro D., Bascunan-Godoy L., Sjölander J., Jurca M., Pin P.A., Nilsson O., Eriksson M.E. (2025). The Circadian Clock of Populus Affects Physiological, Transcriptional and Metabolomic Responses to Osmotic and Ionic Components of Salt Stress. npj Biol. Timing Sleep.

[25] Zhao J.-J., Xiang X., Yang P., Li J., Li H., Wei S.-Y., Wang R.-Q., Wang T., Huang J., Chen L.-H. (2024). Genome-Wide Analysis of C2H2.2 Gene Family in *Populus trichocarpa* and the Function Exploration of PtrC2H2.2-6 in Osmotic Stress. Int. J. Biol. Macromol..

[26] Wang H., Zhao S., Gao Y., Yang J. (2017). Characterization of Dof Transcription Factors and Their Responses to Osmotic Stress in Poplar (*Populus trichocarpa*). PLoS ONE.

[27] Li H.-G., Yang Y., Liu M., Zhu Y., Wang H.-L., Feng C.-H., Niu M.-X., Liu C., Yin W., Xia X. (2022). The In Vivo Performance of a Heat Shock Transcription Factor from *Populus euphratica*, PeHSFA2, Promises a Prospective Strategy to Alleviate Heat Stress Damage in Poplar. Environ. Exp. Bot..

[28] Yang Y., Li H.-G., Wang J., Wang H.-L., He F., Su Y., Zhang Y., Feng C.-H., Niu M., Li Z. (2020). ABF3 Enhances Drought Tolerance via Promoting ABA-Induced Stomatal Closure by Directly Regulating *ADF5* in *Populus euphratica*. J. Exp. Bot..

[29] Guo R., Zhang X., Li M., Zhang H., Wu J., Zhang L., Xiao X., Han M., An N., Xing L. (2023). MdNup62 Involved in Salt and Osmotic Stress Tolerance in Apple. Sci. Rep..

[30] Darriba D., Posada D., Kozlov A.M., Stamatakis A., Morel B., Flouri T. (2020). ModelTest-NG: A New and Scalable Tool for the Selection of DNA and Protein Evolutionary Models. Mol. Biol. Evol..

[31] Kozlov A.M., Darriba D., Flouri T., Morel B., Stamatakis A. (2019). RAxML-NG: A Fast, Scalable and User-Friendly Tool for Maximum Likelihood Phylogenetic Inference. Bioinformatics.

[32] Chen C., Wu Y., Li J., Wang X., Zeng Z., Xu J., Liu Y., Feng J., Chen H., He Y. (2023). TBtools-II: A “One for All, All for One” Bioinformatics Platform for Biological Big-Data Mining. Mol. Plant.

[33] Wei Y., Zhai J., Geng S., Zhang S., Zhao Y., Cui B., Shan H., Li Y., Wang C., Li P. (2025). Genome-Wide Identification and Functional Analysis of TCX Gene Family and the Critical Role of *GhTCX17* in Response to Drought and Salt Stress in Cotton. Funct. Integr. Genom..

[34] Li H.-G., Yang L., Fang Y., Wang G., Lyu S., Deng S. (2025). A Genome-Wide-Level Insight into the HSF Gene Family of *Rhodomyrtus tomentosa* and the Functional Divergence of RtHSFA2a and RtHSFA2b in Thermal Adaptation. Plant Physiol. Biochem..

[35] Qiao X., Li Q., Yin H., Qi K., Li L., Wang R., Zhang S., Paterson A.H. (2019). Gene Duplication and Evolution in Recurring Polyploidization-Diploidization Cycles in Plants. Genome Biol..

[36] Lescot M., Déhais P., Thijs G., Marchal K., Moreau Y., Van de Peer Y., Rouzé P., Rombauts S. (2002). PlantCARE, a Database of Plant *Cis*-Acting Regulatory Elements and a Portal to Tools for in Silico Analysis of Promoter Sequences. Nucleic Acids Res..

[37] Yang Y., Li H.-G., Liu M., Wang H.-L., Yang Q., Yan D.-H., Zhang Y., Li Z., Feng C.-H., Niu M. (2022). PeTGA1 Enhances Disease Resistance against *Colletotrichum gloeosporioides* through Directly Regulating PeSARD1 in Poplar. Int. J. Biol. Macromol..

[38] Su Y., Li H.G., Wang Y., Li S., Wang H.L., Yu L., He F., Yang Y., Feng C.H., Shuai P. (2018). Poplar miR472a Targeting NBS-LRRs Is Involved in Effective Defence against the Necrotrophic Fungus *Cytospora chrysosperma*. J. Exp. Bot..

[39] Liu X., Bao Y., Zhang M.-Y., Zhang H., Niu M.-X., Liu S.-J., Liu M.-Y., Huang M.-B., Liu C., Yin W. (2025). SC35-Mediated bZIP49 Splicing Regulates K^+^ Channel AKT1 for Salt Stress Adaptation in Poplar. Nat. Commun..

[40] Xiang Z.-X., Li W., Lu Y.-T., Yuan T.-T. (2023). Hydrogen Sulfide Alleviates Osmotic Stress-Induced Root Growth Inhibition by Promoting Auxin Homeostasis. Plant J..

[41] He F., Niu M.-X., Wang T., Li J.-L., Shi Y.-J., Zhao J.-J., Li H., Xiang X., Yang P., Wei S.-Y. (2024). The Ubiquitin E3 Ligase RZFP1 Affects Drought Tolerance in Poplar by Mediating the Degradation of the Protein Phosphatase PP2C-9. Plant Physiol..

[42] Moore R.C., Purugganan M.D. (2003). The Early Stages of Duplicate Gene Evolution. Proc. Natl. Acad. Sci. USA.

[43] Fan Y., Cui Q., Li S., Li Y., Yi G., Wang C., Liu Q., Zhang J., Rao G. (2025). Genome-Wide Identification of Phenylacetaldehyde Reductase Genes and Molecular Docking Simulation Study of *OePAR1* in Olives. Forests.

[44] Sun Y., Shi M., Wang D., Gong Y., Sha Q., Lv P., Yang J., Chu P., Guo S. (2023). Research Progress on the Roles of Actin-Depolymerizing Factor in Plant Stress Responses. Front. Plant Sci..

[45] Roy-Zokan E.M., Dyer K.A., Meagher R.B. (2015). Phylogenetic Patterns of Codon Evolution in the ACTIN-DEPOLYMERIZING FACTOR/COFILIN (ADF/CFL) Gene Family. PLoS ONE.

[46] Khatun K., Robin A.H.K., Park J.-I., Kim C.K., Lim K.-B., Kim M.-B., Lee D.-J., Nou I.S., Chung M.-Y. (2016). Genome-Wide Identification, Characterization and Expression Profiling of ADF Family Genes in *Solanum lycopersicum* L. Genes.

[47] Shi M., Wang Y., Lv P., Gong Y., Sha Q., Zhao X., Zhou W., Meng L., Han Z., Zhang L. (2025). Genome-Wide Characterization and Expression Analysis of the ADF Gene Family in Response to Salt and Drought Stress in Alfalfa (*Medicago sativa*). Front. Plant Sci..

[48] Panchy N., Lehti-Shiu M., Shiu S.-H. (2016). Evolution of Gene Duplication in Plants. Plant Physiol..

[49] Cannon S.B., Mitra A., Baumgarten A., Young N.D., May G. (2004). The Roles of Segmental and Tandem Gene Duplication in the Evolution of Large Gene Families in *Arabidopsis thaliana*. BMC Plant Biol..

[50] Xu S., Guo Z., Feng X., Shao S., Yang Y., Li J., Zhong C., He Z., Shi S. (2023). Where Whole-Genome Duplication Is Most Beneficial: Adaptation of Mangroves to a Wide Salinity Range between Land and Sea. Mol. Ecol..

[51] Shi T., Zhang X., Hou Y., Jia C., Dan X., Zhang Y., Jiang Y., Lai Q., Feng J., Feng J. (2024). The Super-Pangenome of *Populus* Unveils Genomic Facets for Its Adaptation and Diversification in Widespread Forest Trees. Mol. Plant.

[52] Xu K., Zhao Y., Zhao S., Liu H., Wang W., Zhang S., Yang X. (2021). Genome-Wide Identification and Low Temperature Responsive Pattern of Actin Depolymerizing Factor (ADF) Gene Family in Wheat (*Triticum aestivum* L.). Front. Plant Sci..

[53] Hou J., Ye N., Dong Z., Lu M., Li L., Yin T. (2016). Major Chromosomal Rearrangements Distinguish Willow and Poplar After the Ancestral “Salicoid” Genome Duplication. Genome Biol. Evol..

[54] Aerts N., Pereira Mendes M., Van Wees S.C.M. (2021). Multiple Levels of Crosstalk in Hormone Networks Regulating Plant Defense. Plant J..

[55] Peng L., He J., Yao H., Yu Q., Zhang Q., Li K., Huang Y., Chen L., Li X., Yang Y. (2022). CARK3-Mediated ADF4 Regulates Hypocotyl Elongation and Soil Drought Stress in *Arabidopsis*. Front. Plant Sci..

[56] Sengupta S., Mangu V., Sanchez L., Bedre R., Joshi R., Rajasekaran K., Baisakh N. (2019). An Actin-Depolymerizing Factor from the Halophyte Smooth Cordgrass, *Spartina alterniflora* (SaADF2), Is Superior to Its Rice Homolog (OsADF2) in Conferring Drought and Salt Tolerance When Constitutively Overexpressed in Rice. Plant Biotechnol. J..

[57] Bi S., Li M., Liu C., Liu X., Cheng J., Wang L., Wang J., Lv Y., He M., Cheng X. (2022). Actin Depolymerizing Factor ADF7 Inhibits Actin Bundling Protein VILLIN1 to Regulate Root Hair Formation in Response to Osmotic Stress in *Arabidopsis*. PLoS Genet..

[58] Dong C.-H., Kost B., Xia G., Chua N.-H. (2001). Molecular Identification and Characterization of the Arabidopsis *AtADF1*, *AtADF5* and *AtADF6* Genes. Plant Mol. Biol..

[59] Wang W., Wen Y., Berkey R., Xiao S. (2009). Specific Targeting of the Arabidopsis Resistance Protein RPW8.2 to the Interfacial Membrane Encasing the Fungal Haustorium Renders Broad-Spectrum Resistance to Powdery Mildew. Plant Cell.

[60] Sun Y., Zhong M., Li Y., Zhang R., Su L., Xia G., Wang H. (2021). GhADF6-Mediated Actin Reorganization Is Associated with Defence against Verticillium Dahliae Infection in Cotton. Mol. Plant Pathol..

[61] Zhu J., Nan Q., Qin T., Qian D., Mao T., Yuan S., Wu X., Niu Y., Bai Q., An L. (2017). Higher-Ordered Actin Structures Remodeled by *Arabidopsis* ACTIN-DEPOLYMERIZING FACTOR5 Are Important for Pollen Germination and Pollen Tube Growth. Mol. Plant.

[62] Qiu T., Su Y., Guo N., Zhang X., Jia P., Mao T., Wang X. (2024). MYB52 Negatively Regulates ADF9-Meditated Actin Filament Bundling in *Arabidopsis* Pavement Cell Morphogenesis. J. Integr. Plant Biol..

[63] Qian D., Zhang Z., He J., Zhang P., Ou X., Li T., Niu L., Nan Q., Niu Y., He W. (2019). *Arabidopsis* ADF5 Promotes Stomatal Closure by Regulating Actin Cytoskeleton Remodeling in Response to ABA and Drought Stress. J. Exp. Bot..

