# Supplementary material for: Systematic Analysis of the Populus ADF Gene Family and the Expression Patterns Under Osmotic Stress
Source: Life (Basel). 2026 May 11;16(5):800. doi: 10.3390/life16050800 (PMC13208785; doi:10.3390/life16050800)
Supplement: Supplementary file 1 [file life-16-00800-s001.zip › Table S1 and Figure S1.pdf]

Table S1: Primers used in this study

gene name	forward primer	reverse primer
18SrRNA	TCAACTTTCGATGGTAGGATAGTG	CCGTGTCAGGATTGGGTAATTT
qADF3	ATTGGTAACTGTGGACAAGGTTGGT	GCTCTGATTCTCGATGCTGTTGGA
qADF6	GGTGTCCTTCAACCTCTCGAATCC	ATCCATCTCTGTAGGGTCAGTAGCC
qADF7	GGCAAACGCAGCATCTGGGAT	GGCACTCATCAGCAGGCAGACT
qADF8	AAGGAGGTTGTGGTGGAGAAGACT	ACGGATTCGAGAGGTTGAAGGAGA
qADF11	GCTATGCTGATCTTGCTGCTTCATT	TGCTCTGATTCTTGATGCTGTTGGA

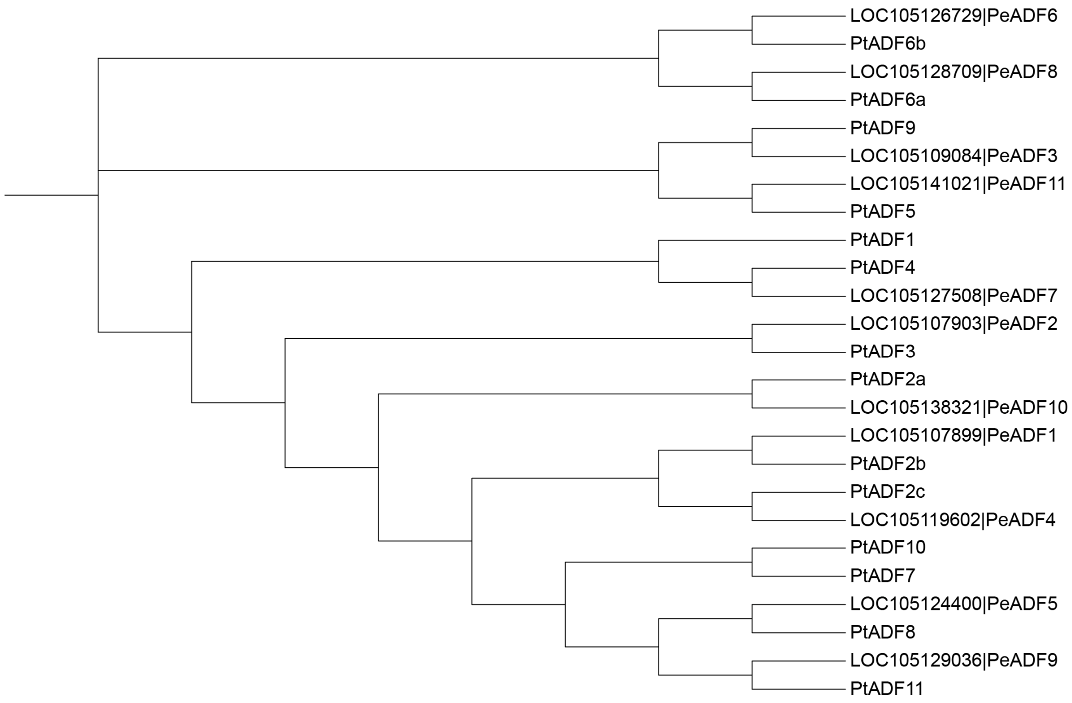

Figure S1: Phylogenetic analysis of *ADF* family genes in *Populus trichocarpa* and *P. euphratica*.
